# Picturing perspectives: development of perspective-taking abilities in 4- to 8-year-olds

**DOI:** 10.3389/fpsyg.2014.00386

**Published:** 2014-04-30

**Authors:** Andrea Frick, Wenke Möhring, Nora S. Newcombe

**Affiliations:** ^1^Department of Psychology, Temple UniversityPhiladelphia, PA, USA; ^2^Department of Psychology, University of BernBern, Switzerland

**Keywords:** cognitive development, spatial cognition, perspective taking, child, viewpoint, reference frame

## Abstract

Although the development of perspective taking has been well researched, there is no uniform methodology for assessing this ability across a wide age span when frames of reference conflict. To address this gap, we created scenes of toy photographers taking pictures of layouts of objects from different angles, and presented them to 4- to 8-year-olds (*N* = 80). Children were asked to choose which one of four pictures could have been taken from a specific viewpoint. Results showed that this new technique confirmed the classic pattern of developmental progress on this kind of spatial skill: (1) 4-year-olds responded near chance level, regardless of layout complexity, (2) there was a growing ability to inhibit egocentric choices around age 6 with layouts of low complexity (one object), (3) performance increased and egocentric responses decreased dramatically around age 7, (4) even at age 8, children still showed considerable individual variability. This perspective taking task can thus be used to address important questions about the supports for early spatial development and the structure of early intellect.

## INTRODUCTION

Visual perspective taking, or the ability to mentally represent a viewpoint different from one’s own, has been extensively studied in the developmental literature, beginning with Piaget and Inhelder’s (1956) seminal work on *The child’s conception of space*. In their *Three Mountains Task*, Piaget and Inhelder asked children to look at a model display of three mountains and to indicate how an observer would see this layout from another position. Up to the age of 9 or 10 years, children made many errors on this task and often picked their own view instead of the observer’s view, thus committing egocentric errors. Piaget and Inhelder argued that young children were unable to coordinate multiple perspectives and they interpreted these difficulties as being indicative of a lack of understanding of what they called projective space. This work inspired hundreds of studies, many of which focused on demonstrating success earlier than 9 or 10 years (see review by Newcombe, 1989).

One important distinction in understanding the development of perspective taking was proposed by Flavell et al. (1981) and Masangkay et al. (1974). These researchers pointed out that knowledge about which objects are visible at all from another viewpoint appears quite early in life. They called this type of non-egocentric inference Level 1 knowledge. Thus, a child at Level 1 knows *that* another person may currently see an object that is not visible for the child, or vice versa. However, computing exactly *how* the other person perceives things is not possible until Level 2. In support of this distinction, recent studies have demonstrated Level 1 abilities even in infancy (Moll and Tomasello, 2006; Sodian et al., 2007). In contrast, Level 2 rules are first evident at around 4 or 5 years of age (Masangkay et al., 1974; Flavell et al., 1980; Pillow and Flavell, 1986) but performance improves considerably between age 6 and 8 (Salatas and Flavell, 1976).

Despite a large body of research on perspective taking, our knowledge of the individual and task factors affecting the emergence of Level 2 perspective taking is still very limited. One relevant factor was suggested by Fishbein et al. (1972), who found that children from 3–9 years performed better in picture selection tasks if only one object was involved, compared to when an array of three objects was presented (see also Gzesh and Surber, 1985). Tasks with only one object may be spatially less complex as no array-internal relations have to be encoded and coordinated with the position (or line of sight) of the observer. Consistent with this notion, Ives (1980) found that responses of children as young as 3 years old were about 90% correct if they were asked whether they would see the front, side, or back of a single fronted object (e.g., a horse) from a specified position. However, several other studies with children from kindergarten to 6th grade have failed to find an effect of number of objects in the array (Brodzinsky et al., 1972; Minnigerode and Carey, 1974; Nigl and Fishbein, 1974).

Another important factor affecting the emergence of perspective taking lies in the nature of the dependent variable and whether it requires the suppression of conflicting frames of reference. Piaget and Inhelder (1956) used picture selection or model building tasks, in which children sat within the frame of reference of a room while attempting to imagine an array from a different vantage point. Huttenlocher and Presson (1973, 1979) found that 8- or 9-year-olds did much better when asked about particular items from a different vantage point (e.g., if you sat over there, what would be closest to you?), and argued that such questions were easier because they suggested a frame of reference other than the surrounding room (i.e., the child’s body in an imagined position served as the reference point). Even preschoolers succeed on tasks in which responses are not influenced by conflicting frames of reference (Newcombe and Huttenlocher, 1992). Importantly, however, such questions are not easier in general; they are harder when children are asked to imagine the array rotating (Huttenlocher and Presson, 1973, 1979). The contrast between array rotation and perspective taking and its interaction with the processing demands of the dependent variable have been confirmed in many subsequent studies (Simons and Wang, 1998; Wang and Simons, 1999; Wraga et al., 2000; Hegarty and Waller, 2004; Nardini et al., 2006).

Taken together, a great deal of research tells us that, on tasks that minimize spatial and representational complexity, the basic ability to take someone else’s perspective is present from infancy, and may provide the foundation for social cognitive abilities, such as theory of mind (Sodian et al., 2007). However, from a spatial cognition point of view, the spatially rich Level 2 perspective-taking tasks with conflicting frames of reference are interesting because they concern the ability to represent and coordinate multiple perspectives in one coherent spatial framework. This ability may be ecologically meaningful and predictive of real-world spatial performance, as suggested by research in adults (Hegarty and Waller, 2004, 2005). For example, Hegarty and Waller (2004) found that perspective taking was correlated with participants’ self-reported sense of direction, which in turn had predictive validity as a measure of spatial cognition in large-scale environments (Sholl, 1988; Hegarty et al., 2002). One of the very few developmental studies showed that perspective taking (measured by a modified Three Mountains Task) correlated with performance in a mapping task (Liben and Downs, 1993).

One reason why individual differences in Level 2 perspective taking and their correlates are little investigated in children may be that there is no simple and easy-to-administer test of the ability. Many existing paradigms were not applied below age 6 (Brodzinsky et al., 1972; Liben and Downs, 1993; Surtees and Apperly, 2012). The ones that are suitable for younger children require complex setups involving farm scenes, toy houses, dolls, toy boats, blocks, or papier-mâché mountains, (Coie et al., 1973; Minnigerode and Carey, 1974; Ives, 1980; Liben and Belknap, 1981; Brodzinsky, 1982; Gzesh and Surber, 1985). Such three-dimensional setups are hard to reproduce in replication studies and cumbersome to haul to schools in large-scale studies. Furthermore, some of the previous tasks required children to understand or even produce complex spatial terms in describing how displays looked to them (e.g., “right side up”) or other observers (“upside down”; e.g., Masangkay et al., 1974).

In the present study, we thus investigated the development of Level 2 perspective taking in children from 4–8 years, using a task similar to the original Three Mountains Task in terms of spatial and representational complexity. However, in the present task, spatial layouts were presented on paper, which made the task easier to administer than using a three-dimensional display or model. The general format of the present task was based on a study with adult participants (Hegarty and Waller, 2004). In this task, computer graphics showed three photographers taking pictures of a complex three-dimensional layout of objects. Different pictures of the layout were presented and adults were asked to decide which of the photographers (or none) could have taken the picture. As this task was hard even for adults, a simplified version was created for the age groups of the present study. We presented scenes with toy photographers taking pictures of different layouts of objects and asked children to choose which one of four pictures was most likely taken by a specific photographer. Most notably, our task differed from Hegarty and Waller’s in that we used photographs of Playmobil figures rather than computer graphics, and the choice alternatives showed perspectives that were easier to differentiate as they differed by larger angles (90°).

Children from 4–8 years were tested, based on previous work showing that Level 2 knowledge emerges around 4 or 5 years of age (Masangkay et al., 1974; Flavell et al., 1980; Pillow and Flavell, 1986), and improves considerably between age 6 and 8 (Salatas and Flavell, 1976). We investigated Level 2 perspective taking abilities in this age range using a single methodology, and also tested effects of different angular disparities between observer and target perspectives. Given that previous studies led to mixed results about effects of spatial complexity of presented layouts, we also systematically examined effects of layout complexity. We expected performance to be highest on trials presenting only one fronted object, lower performance if two symmetrical objects were presented and the viewpoint had to be inferred based on their spatial relation, and the lowest performance if arrays of multiple objects were presented for which internal spatial configurations had to be considered.

## MATERIALS AND METHODS

### PARTICIPANTS

Participants were 80 children, with 16 children (8 boys and 8 girls) in each of the following age groups: 4-year-olds (mean age = 4;4, range = 4;0–4;11); 5-year-olds (mean age = 5;6, range = 5;1–5;11); 6-year-olds (mean age = 6;4, range = 6;0–6;11); 7-year-olds (mean age = 7;6, range = 7;1–7;11); 8-year-olds (mean age = 8;7, range = 8;1–9;0). Two additional children (one 4- and one 5-year-old) were tested but excluded from analyses due to incorrect responses on one of the three criterion trials (see next section). Children were recruited from a database of families who had volunteered to participate in developmental research. They were predominantly Caucasian, from middle-class backgrounds, and lived in urban and suburban areas of a large U.S. city. All parents provided written informed consent and all children provided verbal assent. All procedures used in this research were conducted according to ethical guidelines and approved by the Institutional Review Board at Temple University.

### STIMULUS MATERIAL AND PROCEDURE

**FIGURE 1:**
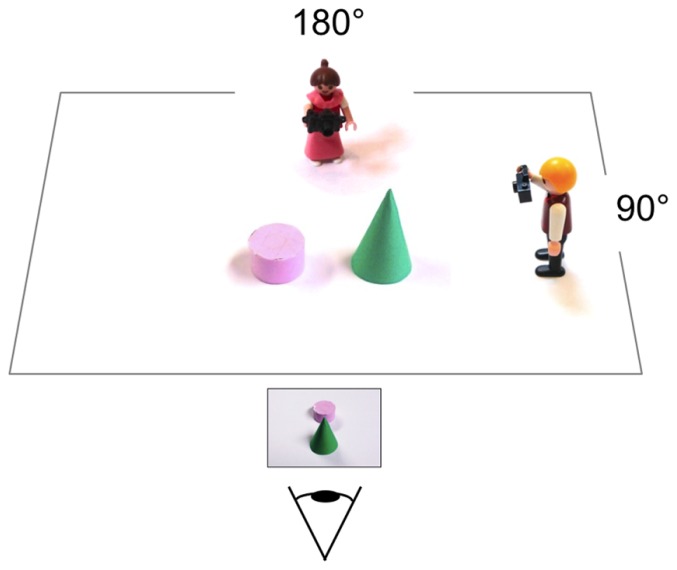
**Schematic of the layout and stimuli presented in the instruction phase.** The symbolic eye indicates the children’s viewing position.

Participants were tested in a laboratory room. In four instruction trials, real three-dimensional objects and photographers were presented (**Figure 1**). A cone and a cylinder were presented side-by-side on a letter-size white cardboard and placed on a table. Two Playmobil figures were placed on the cardboard and introduced as Lisa and Peter. Both figures were holding a camera, pointing it at the objects, one at 90° and one at 180° angular difference to the child’s line of sight. In each of the four practice trials, a picture of the cone and cylinder was presented that was taken from a different perspective. Children were asked whether they thought that Lisa or Peter (or no one) has taken the picture. After the children gave their responses (verbally), the experimenter encouraged them to walk behind Lisa and Peter and peek over their shoulders to check. One picture showed the child’s own perspective so neither of the photographers could have taken it. In this case, the experimenter encouraged the child to return to its seat after checking, and pointed out that neither Lisa nor Peter could have taken this picture because it was taken from where the child was sitting.

A three-dimensional setup was chosen for the instruction trials, as pilot experiments showed that young children had difficulties understanding the instructions if they were not allowed to physically move behind the photographers to check their answers. Furthermore, a setup with two photographers was used in instruction trials (in analogy to the original task by Hegarty and Waller, 2004), to highlight the fact that different positions of photographers afford different perspectives. Moreover, previous work had shown that children are more likely to understand the task if a doll was used in the instruction (Newcombe and Huttenlocher, 1992).

Subsequent trials were presented as color printouts in letter-sized transparent document pockets in a binder. The first two trials served to ease the transition from the three-dimensional instruction trials. These trials showed two photographers at 0, 90, or 180° angular difference from the child’s perspective, and children had to guess which photographer could have taken one particular picture. The next three trials showed only one photographer with four alternative pictures, and children were asked which picture this photographer could have taken. These three trials served as criterion trials and were very easy, as the four alternatives each showed completely different objects, only one of which was in front of the photographer’s camera. Thus, following the photographer’s line of sight (or Level I perspective taking) was sufficient to succeed on these trials.

**FIGURE 2:**
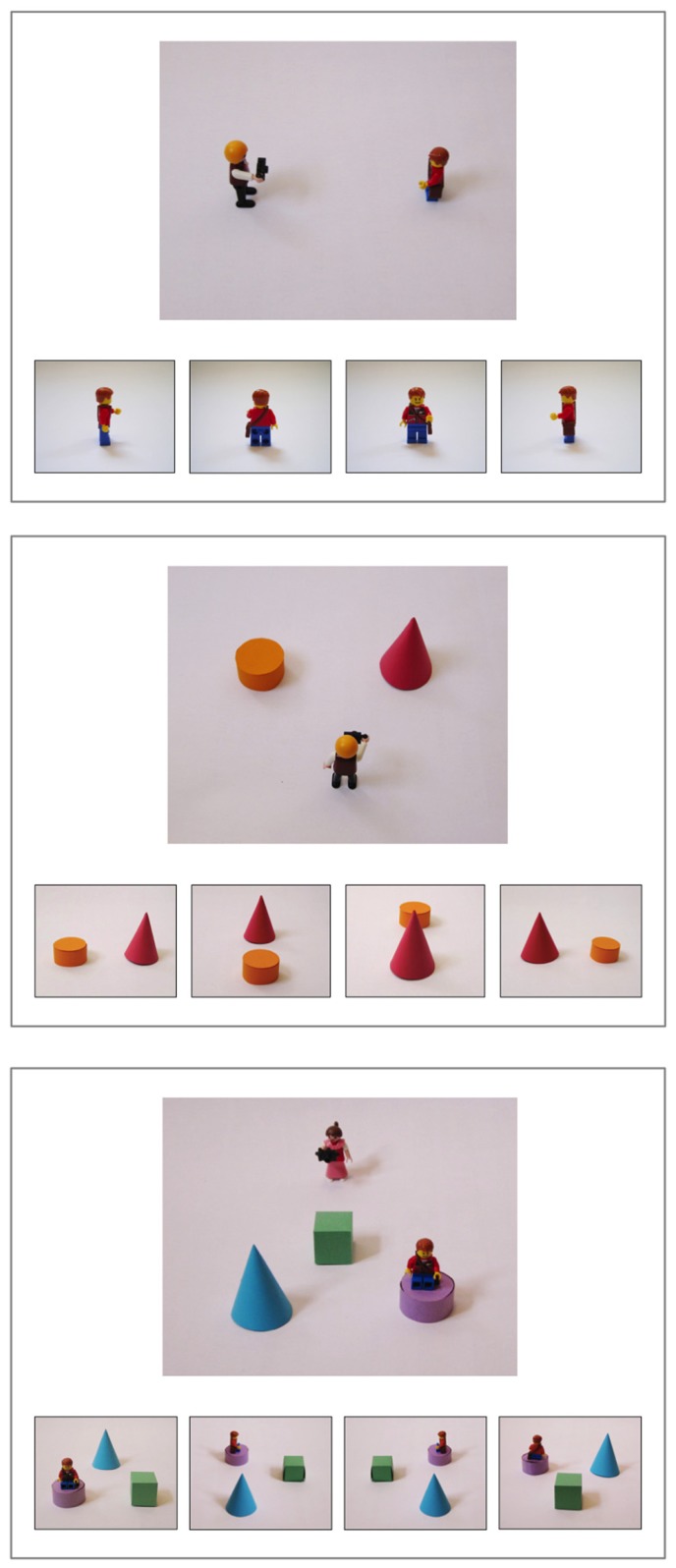
**Examples for stimuli presented in test trials, varying in spatial complexity (1, 2, or 4 objects) and perspectives (90, 0, or 180°).** Stimuli were presented exactly as shown, as color printouts on separate letter-sized sheets of paper.

The subsequent test trials presented one photographer and four choice alternatives, but now all alternatives showed the same object(s) from different perspectives (see **Figure 2**). The choice alternatives were distributed horizontally below the layout picture. One of the alternatives showed the correct view and three were foils, in which the orientation or spatial relations among the objects would not match the photographer’s perspective. Whereas on 0° trials the child’s own perspective was the correct answer, on 90 and 180° trials the child’s perspective served as one of the foils. The layouts consisted of one, two, or four objects. To clearly distinguish the views on one-object trials, a fronted asymmetric object was used: a figurine with a face, a bag over one shoulder, and one arm raised. On four-object trials, roughly half of the foils also differed regarding the internal spatial relations between objects.

All layout pictures were taken from an oblique angle (camera slanted by ca. 45°), similar to how a layout on the table would look from the child’s eye level (e.g., in instruction trials). Thus, all objects in the array were clearly visible and none of them was occluded. The choice pictures were taken from a slightly lower camera position, so that the 0° choice alternative was similar but not identical to the layout picture (and the child’s perspective).

Children were instructed to “point to the picture Lisa (Peter) has taken from where she (he) is standing.” Children were also encouraged to “pretend peeking over her (his) shoulder to find out.” If a child pointed ambiguously, the experimenter asked the child to point again because she did not clearly see which one the child had chosen. If a child pointed to two pictures in a row the child was asked again which one was the final answer. The experimenter wrote down the response and flipped the page for the next trial. There was no time limit to complete a trial and the whole experiment took about 5 to 7 min.

### DESIGN

Test trials varied in the angular difference between the photographer’s and the child’s perspectives (0, 90, or 180°) and complexity (1, 2, or 4 objects in the layout). Each of these combinations was presented twice, with different objects and a different photographer, amounting to a total of 18 test trials. The number of objects in the layout was blocked, such that complexity increased from block to block. However, half of the children of each age group and sex were presented with the reverse trial order, such that complexity decreased from block to block, in order to rule out confounding effects of fatigue or practice. The different angles were presented in a predetermined quasi-random order, with the restriction that no angle was presented twice in a row. Photographers (Lisa or Peter) and objects (colors, spatial configurations) were also changed between trials, in order to make the task more entertaining and prevent children from trying to remember their answers in previous trials. The position of the correct choice was quasi-randomized across trials. A trial was scored with one point if the correct picture was selected, and with zero points if any of the three foils were chosen, thus a total score of 18 could be reached.

## RESULTS

### INTERNAL CONSISTENCY

We calculated Guttman’s split-half coefficient (i.e., the correlation between trials analogous in terms of angle and complexity, but with different objects and photographers), which showed an excellent internal consistency of 0.91.

### ABOVE CHANCE PERFORMANCE

In a first analysis, performance was classified according to whether or not it exceeded chance, that is, whether children solved 9 trials or more out of 18 trails correctly (Binomial distribution, *p* < 0.05). The number of children who performed above chance increased with age, with significant improvements from 5 to 6 and from 7 to 8 years of age (see **Table 1**, first column; asterisks indicate significant chi-square statistics showing more above-chance performing children than in the previous age group).

**Table 1:** Number of children who performed above chance on the individual level, and mean accuracy (percentage, standard deviation, and range) per age group.

	Above chance performers	Accuracy		
		%	*SD*	Range
4-year-olds	2	35	15	11–72
5-year-olds	3	39	9	22–56
6-year-olds	9*	50	12	33–78
7-year-olds	9	51	17	22–83
8-year-olds	14*	76*	21	39–100

*Asterisks indicate a significant difference compared to the previous age group (*p* < 0.05) in chi-square tests (second column) and Sidak-corrected *post hoc* tests (third column).*

### MEAN ACCURACY

To test for effects of spatial complexity and angular difference between the photographer’s and the child’s perspectives on the proportion of correctly solved trials, data were averaged across measurement repetitions. A preliminary ANOVA was calculated with angle (0, 90, 180°), complexity (1, 2, 4 objects), age group (5), sex (2), and trial order (forward vs. backward) as independent variables, and accuracy as dependent variable. The analysis showed no significant main effects of or interactions with trial order (all *F*s < 2.02, all *p*s > 0.05). This suggests that there were no significant effects due to practice or fatigue; therefore this variable was not included in subsequent analyses.

An ANOVA was calculated with angle (0, 90, 180°), complexity (1, 2, 4 objects), age group (5), and sex (2) as independent variables and accuracy as dependent variable. Results showed significant main effects of age group, *F*(4,70) = 16.04, *p* < 0.001, η^2^ = 0.48. *Post hoc* tests (Sidak corrected here, and throughout) showed that there was a dramatic increase in correct choices especially between 7 and 8 years of age (from 51 to 76%, *p* < 0.001, see **Table 1**). The group of 8-year-olds differed from all other age groups (all *p*s < 0.001), whereas age groups younger than 8 years did not differ from each other (all *p*s > 0.08). However, even at age 8, there was still considerable variability in individual test scores (see **Table 1** for standard deviations).

Furthermore, there was a significant main effect of angle, *F*(2,140) = 191.24, *p* < 0.001, η^2^ = 0.73, and an interaction of age group and angle, *F*(8,140) = 4.56, *p* < 0.001, η^2^ = 0.21. *Post hoc* tests investigating this interaction showed that 4-year-olds differed from all other age groups on 0° trials (all *p*s < 0.01). On the other hand, 8-year-olds differed from all other age groups on 90 and 180° trials (all *p*s < 0.05). Other age groups did not differ significantly at any angle (all *p*s > 0.44). An interaction between sex and angle, *F*(2,140) = 4.76, *p* < 0.05, η^2^ = 0.06, was due to males outperforming females on 0° trials that did not require a perspective change (*p* < 0.05), whereas on 90 and 180° trials there were no sex differences (*p*s > 0.11).

**FIGURE 3:**
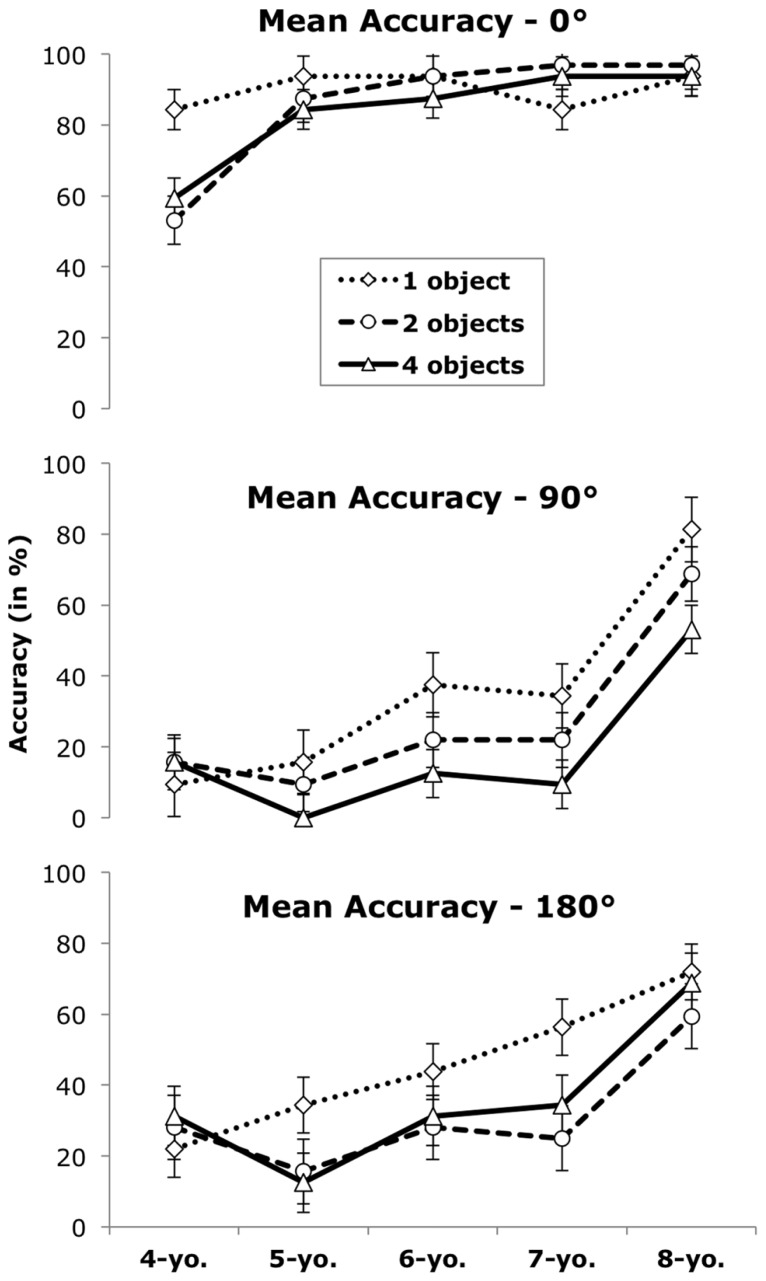
**Mean correct responses (in %) by age group and number of objects per angle.** Error bars indicate the standard error of the mean.

A main effect of complexity, *F*(2,140) = 13.21, *p* < 0.001, η^2^ = 0.16, was qualified by a two-way interaction between complexity and angle, *F*(4,280) = 2.65, *p* < 0.05, η^2^ = 0.04, and a three-way interaction of complexity, angle, and age group, *F*(16,280) = 2.59, *p* < 0.01, η^2^ = 0.13 (see **Figure 3**). *Post hoc* tests showed that complexity mattered mostly on 90° trials (all *p*s < 0.05) and 180° trials (one object significantly better than two, *p* < 0.05). On the other hand, on trials requiring no perspective change, the number of objects did not significantly affect accuracy (all *p*s > 0.11) and performance was high overall (87% accuracy). However, the three-way interaction with age group indicates that this was not true for 4-year-olds, who performed better on 0° trials when only one object was involved (84% accuracy) as compared to multiple objects (*p*s < 0.05). In addition, they performed poorly on trials that required a perspective change (from 9–31% accuracy) regardless of complexity (*p*s > 0.67). The ANOVA showed no other significant effects (all *F*s < 1.09, *p*s > 0.36).

### EGOCENTRIC ERRORS

Finally, children’s responses were analyzed regarding their tendency to choose the alternative showing their own perspective. Such an egocentric response is the correct solution on 0° trials, but incorrect on 90 and 180° trials. Therefore, only 90 and 180° trials were included in this analysis. An ANOVA was calculated with angle (90, 180°), complexity (1, 2, 4 objects), age group (5), and sex (2) as independent variables and proportion of egocentric responses as dependent variable.

**FIGURE 4:**
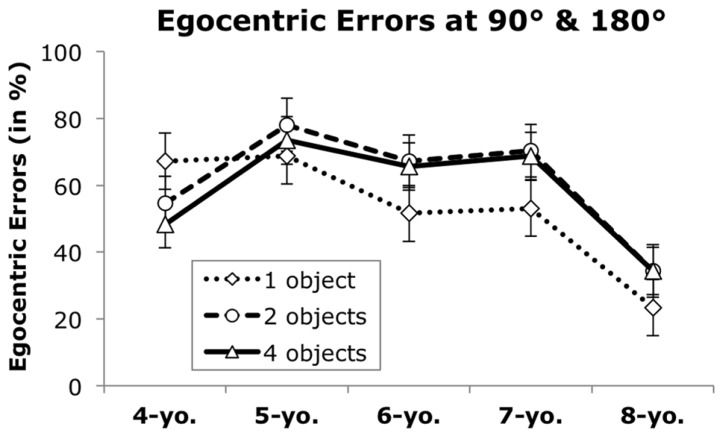
**Mean egocentric errors (in %) in trials requiring a perspective change, by age group and number of objects.** Error bars indicate the standard error of the mean.

The analysis yielded significant main effects of age group, *F*(4,70) = 5.80, *p* < 0.001, η^2^ = 0.25, and complexity, *F*(2,140) = 3.40, *p* < 0.05, η^2^ = 0.05, and a significant interaction between age group and complexity, *F*(8,140) = 2.32, *p* < 0.05, η^2^ = 0.12. **Figure 4** shows that, especially when multiple objects were involved, egocentric errors increased from 4 to 5 years of age, but generally decreased after 5 years of age. *Post hoc* tests suggested that 5-, 6-, and 7-year-olds chose the egocentric view significantly more often than 8-year-olds when multiple objects were involved (*p*s < 0.05). However, when only one object was in the layout, 6- and 7-year-olds did not differ from 8-year-olds (*p*s > 0.13). A main effect of sex, *F*(1,70) = 5.00, *p* < 0.05, η^2^ = 0.07, indicated that boys chose the egocentric perspective more often than girls (64 and 51%, respectively). The ANOVA yielded no other significant effects, (all *F*s < 2.21, *p*s > 0.11).

## DISCUSSION

In the present study, we investigated 4- to 8-year-old children’s ability to represent and coordinate multiple perspectives when dealing with conflicting frames of reference. We presented pictures showing scenes with a toy photographer taking pictures of a single fronted object or multiple objects from different angles. Children were asked to select the toy photographer’s perspective among four photographs. Results showed an increase in correct choices with age, with the most dramatic improvement between 7 and 8 years of age. The number of children who performed above chance level also increased with age, with significant improvement from 5 to 6 and from 7 to 8 years of age. 

A more detailed analysis of children’s incorrect responses showed that when multiple objects were involved, egocentric errors increased from 4 to 5 years of age. In combination with the low accuracy observed in 4-year-olds, it appears that 4-year-olds may have responded randomly, whereas at 5 years of age children began to more systematically choose their own perspective. Egocentric errors peaked at 5 years of age, followed by a general decrease in this response bias, suggesting a growing ability to inhibit egocentric choices and to rely on allocentric representations of space with increasing age (Lourenco and Frick, 2013). Boys made more egocentric errors than girls, indicating that they chose a view that corresponded to their own perspective more often than girls. This may also explain why boys generally outperformed females on 0° trials, where the egocentric response was the correct choice. However, there were no sex differences in accuracy on trails that required a perspective change.

Even though performance improved with age, there was still considerable variability in individual test scores, as suggested by large standard deviations. Thus, although some 8-year-olds performed almost perfectly, there was still room for improvement for many 8-year-olds, especially for spatially complex layouts showing multiple objects. For all children except the youngest age group, spatial complexity mattered most on trials that required a perspective change, whereas performance on 0°-trials was generally very high. On the other hand, spatial complexity did not affect performance on trials that did not require a perspective change – again with exception of 4-year-olds, whose performance suffered even on 0°-trials when more than one object was involved.

Layouts of low spatial complexity, exhibiting only one fronted object, appeared to be easier for 4-year-olds, and a decrease in egocentric responses was observed about 2 years earlier on such items. These results corroborate previous findings that children succeeded earlier if fewer objects are involved (Fishbein et al., 1972; Gzesh and Surber, 1985). When only one fronted object is presented, success can be achieved by following an observer’s line of sight and noticing whether he would see the front, back, or a particular side of the object. This may have reduced task difficulty, and made the task more similar to a Level 1 perspective taking task.

On the other hand, if multiple objects are involved, spatial relations between the objects in the array have to be considered. Our results suggest that this ability was virtually non-existent in 4-year-olds, and improved considerably from 7 to 8 years of age. These results are consistent with previous literature showing that Level 2 rules are acquired at around 4 or 5 years of age (Masangkay et al., 1974; Flavell et al., 1980; Pillow and Flavell, 1986), and improve considerably between age 6 and 8 (Salatas and Flavell, 1976).

At the same time, our results stand in contrast to claims that younger children can demonstrate Level 2 perspective taking skills. For example, in a study by Liben (1978) that aimed to minimize spatial complexity, the child and/or experimenter wore colored glasses and the child was asked to describe in which color a white card appeared to each of them. At ages 6–7, answers were consistently correct, and even at ages 4–5 more than half the children were able to correctly answer questions about the experimenter’s view. In similar but more recent studies, Moll et al. (2013) and Moll and Meltzoff (2011) showed that children as young as 3 years old knew how an object would look to another person if viewed through a color filter. However, it is not entirely clear whether tasks such as these are essentially Level 1 tasks, as even though they ask a *how*-question, tracing a line of sight shows a path that traverses the filter.

These conflicting results also suggest the need for finer-grained research on the processes underlying the different perspective taking tasks. The classic perspective taking task involves arrays of multiple objects and tests with picture choice and model building. This task probably indexes several different skills, which may include internal coding of the array of targets (i.e., relative distances and positions among multiple objects forming a coherent configuration), possibly using a common framework of landmarks or an external frame of reference. However, if such a frame of reference is used in coding the array, the challenge is suppressing awareness of the physically present frame of reference when responding (Huttenlocher and Newcombe, 1984; Newcombe, 1989). In support of this notion, individual differences in children’s perspective taking (measured by a task similar to the Three Mountains Task) have previously been linked to cognitive styles, such as reflection-impulsivity (Brodzinsky, 1982). Future research should take a closer look at whether and how the development of perspective taking is associated with progress in other spatial and non-spatial cognitive abilities.

Taken together, the present findings showed a growing ability to inhibit egocentric choices and to recognize the correct viewpoint between 5 to 8 years of age, with the largest change between 7 and 8 years. Using a new paradigm that is easy to administer in school settings and allows for systematic experimental variation of task factors such as layout complexity, the present study showed that an increase in accuracy and a decrease in egocentric responses can be observed earlier when spatial complexity is low. Most 4-year-olds responded near chance level when a change in perspective was required, and large standard deviations in children’s responses suggest that there are large individual differences in this ability at an early age, which may be precursors of the individual differences found in adults (for a review see Hegarty and Waller, 2005). In light of findings that the ability to coordinate perspectives and reference frames bears importance to many spatial tasks in adults (Hegarty and Waller, 2004, 2005), a deeper understanding of the origins and long-term effects of this ability is essential.

## Conflict of Interest Statement

The authors declare that the research was conducted in the absence of any commercial or financial relationships that could be construed as a potential conflict of interest.

## References

[B1] Brodzinsky D. M. (1982). Relationship between cognitive style and cognitive development: a 2-year longitudinal study. *Dev. Psychol.* 18 617–626 10.1037/0012-1649.18.4.617

[B2] Brodzinsky D. M. Jackson J. P. Overton W. F. (1972). Effects of perceptual shielding in the development of spatial perspectives. *Child Dev.* 43 1041–1046 10.2307/1127655 5078989

[B3] Coie J. D. Costanzo P. R. Farnill D. (1973). Specific transitions in the development of spatial perspective-taking ability. *Dev. Psychol.* 9 167–177 10.1037/h0035062

[B4] Fishbein H. D. Lewis S. Keiffer K. (1972). Children’s understanding of spatial relations: coordination of perspectives. *Dev. Psychol.* 7 21–33 10.1037/h0032858

[B5] Flavell J. H. Everett B. A. Croft K. Flavell E. R. (1981). Young children’s knowledge about visual perception: further evidence for the Level 1–Level 2 distinction. *Dev. Psychol.* 17 99–103 10.1037/0012-1649.17.1.99

[B6] Flavell J. H. Flavell E. F. Green F. L. Wilcox S. A. (1980). Young children’s knowledge about visual perception: effect of observer’s distance from target on perceptual clarity of target. *Dev. Psychol.* 16 10–12 10.1037/0012-1649.16.1.10

[B7] Gzesh S. M. Surber C. F. (1985). Visual perspective-taking skills in children. *Child Dev.* 56 1204–1213 10.2307/1130235 4053740

[B8] Hegarty M. Richardson A. E. Montello D. R. Lovelace K. Subbiah I. (2002). Development of a self-report measure of environmental spatial ability. *Intelligence* 30 425–447 10.1016/S0160-2896(02)00116-2

[B9] Hegarty M. Waller D. (2004). A dissociation between mental rotation and perspective-taking spatial abilities. *Intelligence* 32 175–191 10.1016/j.intell.2003.12.001

[B10] Hegarty M. Waller D. (2005). “Individual differences in spatial abilities,” in *The Cambridge Handbook of Visuospatial Thinking* eds Shah P. Miyake A. (Cambridge: Cambridge University Press) 121–169 10.1017/CBO9780511610448.005

[B11] Huttenlocher J. Newcombe N. (1984). “The child’s representation of information about location,” in *Origins of Cognitive Skills* ed. Sophian C. (Hillsdale, NJ: Lawrence Erlbaum Associates) 81–111

[B12] Huttenlocher J. Presson C. C. (1973). Mental rotation and the perspective problem. *Cogn. Psychol.* 4 277–299 10.1016/0010-0285(73)90015-7

[B13] Huttenlocher J. Presson C. C. (1979). The coding and transformation of spatial information. *Cogn. Psychol.* 11 375–394 10.1016/0010-0285(79)90017-3 519969

[B14] Ives W. (1980). Preschool children’s ability to coordinate spatial perspectives through language and pictures. *Child Dev.* 51 1303–1306 10.2307/1129579 7471929

[B15] Liben L. S. (1978). Perspective-taking skills in young children: seeing the world through rose-colored glasses. *Dev. Psychol.* 14 87–92 10.1037/0012-1649.14.1.87

[B16] Liben L. S. Belknap B. (1981). Intellectual realism: implications for investigations of perceptual perspective taking in young children. *Child Dev.* 52 921–924 10.2307/1129095 7285661

[B17] Liben L. S. Downs R. M. (1993). Understanding person-space-map relations: cartographic and developmental perspectives. *Dev. Psychol.* 29 739–752 10.1037/0012-1649.29.4.739

[B18] Lourenco S. F. Frick A. (2013). “Remembering where: the origins and early development of spatial memory,” in *Wiley-Blackwell Handbook on the Development of Children’s Memory* eds Bauer P. J. Fivush R. (Chichester: John Wilsey & Sons Ltd) 10.1002/9781118597705.ch16

[B19] Masangkay Z. S. Mccluskey K. A. Mcintyre C. W. Sims-Knight J. Vaughn B. E. Flavell J. H. (1974). The early development of inferences about the visual percepts of others. *Child Dev.* 45 357–366 10.2307/1127956 4837714

[B20] Minnigerode F. A. Carey R. N. (1974). Development of mechanisms underlying spatial perspectives. *Child Dev.* 45 496–498 10.2307/1127976 4837720

[B21] Moll H. Meltzoff A. N. (2011). How does it look? Level 2 perspective-taking at 36 months of age. *Child Dev.* 82 661–673 10.1111/j.1467-8624.2010.01571.x 21410927

[B22] Moll H. Meltzoff A. N. Merzsch K. Tomasello M. (2013). Taking versus confronting visual perspectives in preschool children. *Dev. Psychol.* 49 646–654 10.1037/a0028633 22612438

[B23] Moll H. Tomasello M. (2006). Level 1 perspective-taking at 24 months of age. *Br. J. Dev. Psychol.* 24 603–613 10.1348/026151005X55370

[B24] Nardini M. Burgess N. Breckenridge K. Atkinson J. (2006). Differential developmental trajectories for egocentric, environmental and intrinsic frames of reference in spatial memory. *Cognition* 101 153–172 10.1016/j.cognition.2005.09.005 16359653

[B25] Newcombe N. (1989). “The development of spatial perspective taking,” in *Advances in Child Development and Behavior* ed. Reese H. W. (San Diego, CA: Academic Press) 203–247 10.1016/s0065-2407(08)60415-2 2688376

[B26] Newcombe N. Huttenlocher J. (1992). Children’s early ability to solve perspective-taking problems. *Dev. Psychol.* 28 635–643 10.1037/0012-1649.28.4.635

[B27] Nigl A. J. Fishbein H. D. (1974). Perception and conception in coordination of perspectives. *Dev. Psychol.* 10 858–866 10.1037/h0037247

[B28] Piaget J. Inhelder B. (1956). *The Child’s Conception of Space*, Trans. Langdon F. J. Lunzer J. L. London: Routledge and Kegan Paul

[B29] Pillow B. H. Flavell J. H. (1986). Young children’s knowledge about visual perception: projective size and shape. *Child Dev.* 57 125–135 10.2307/1130644 3948590

[B30] Salatas H. Flavell J. H. (1976). Perspective taking: the development of two components of knowledge. *Child Dev.* 47 103–109 10.2307/1128288

[B31] Sholl M. J. (1988). The relation between sense of direction and mental geographic updating. *Intelligence* 12 299–314 10.1016/0160-2896(88)90028-1

[B32] Simons D. J. Wang R. F. (1998). Perceiving real-world viewpoint changes. *Psychol. Sci.* 9 315–320 10.1111/1467-9280.00062

[B33] Sodian B. Thoermer C. Metz U. (2007). Now I see it but you don’t: 14-month-olds can represent another person’s visual perspective. *Dev. Sci.* 10 199–204 10.1111/j.1467-7687.2007.00580.x 17286844

[B34] Surtees A. D. Apperly I. A. (2012). Egocentrism and automatic perspective taking in children and adults. *Child Dev.* 83 452–460 10.1111/j.1467-8624.2011.01730.x 22335247

[B35] Wang R. F. Simons D. J. (1999). Active and passive scene recognition across views. *Cognition* 70 191–210 10.1016/S0010-0277(99)00012-8 10349763

[B36] Wraga M. Creem S. H. Proffitt D. R. (2000). Updating displays after imagined object and viewer rotations. *J. Exp. Psychol. Learn. Mem. Cogn*. 26 151–168 10.1037/0278-7393.26.1.151 10682295

